# Newborn screening versus clinical diagnosis: comparison of nutritional status in children with cystic fibrosis – a longitudinal study

**DOI:** 10.1590/1984-0462/2026/44/2025204

**Published:** 2026-07-10

**Authors:** Gabriella Santana Fernandes dos Santos, Carla Hilário da Cunha Daltro, Edna Lúcia D’Souza

**Affiliations:** a Universidade Federal da Bahia, Programa de Pós Graduação em Medicina e Saúde, Salvador, BA, Brasil.

**Keywords:** Cystic fibrosis, Newborn screening, Nutritional status, Pancreatic insufficiency, Growth, Genotype, Fibrose cística, Triagem neonatal, Estado nutricional, Insuficiência pancreática, Crescimento, Genótipo

## Abstract

**Objective:**

The aim of this study was to compare the nutritional status of individuals with cystic fibrosis (CF) diagnosed either through newborn screening (NBS) or based on clinical findings (early vs. late), at the time of diagnosis and 1 year later.

**Methods:**

This was an observational study including CF patients followed at a Referral Center from 2005 to 2023. Participants were classified into three subgroups according to the method and age at diagnosis: 1 (NBS); 2 (early signs and symptoms if the CF diagnosis was before 2 years of age); and 3 (late signs and symptoms if the CF diagnosis was after 2 years of age). Nutritional parameters were compared at diagnosis and 1 year after treatment.

**Results:**

A total of 63 individuals were included, 57.1% male, 81.0% non-white, and median (interquartile range) age at diagnosis of 6 (2–42) months. There were 21, 23, and 19 in subgroups 1, 2, and 3, respectively. At diagnosis, 26 (41.3%) individuals had short stature (SST) or very SST, corresponding to 57.1, 43.5, and 21.1% of children in subgroups 1, 2, and 3, respectively. After 1 year, these proportions decreased to 28.5 and 30.4% in subgroups 1 and 2, while remaining unchanged in subgroup 3. Pancreatic insufficiency was present in 85.7, 69.6, and 68.4% of participants in subgroups 1, 2, and 3, respectively.

**Conclusions:**

Short stature was frequent at diagnosis, even among children identified by NBS. After 1 year of follow-up, height improved in subgroups 1 and 2 but remained unchanged in subgroup 3. Early diagnosis and timely nutritional interventions are essential to prevent and correct growth deficits in individuals with CF.

## RESUMO

**Objetivo:**

Comparar o estado nutricional de indivíduos com fibrose cística (FC) diagnosticados por triagem neonatal (TN) ou com base em achados clínicos (precoce vs. tardio), no momento do diagnóstico e um ano depois.

**Métodos:**

Estudo observacional com pacientes com FC acompanhados em um centro de referência de 2005 a 2023. Os participantes foram classificados em três subgrupos, de acordo com o método e a idade ao diagnóstico: 1 (TN); 2 (sinais e sintomas precoces, se o diagnóstico de FC ocorreu antes dos dois anos de idade) e 3 (sinais e sintomas tardios, se o diagnóstico de FC ocorreu após os dois anos de idade). Os parâmetros nutricionais foram comparados no momento do diagnóstico e um ano após o tratamento.

**Resultados:**

O total de 63 indivíduos foi incluído, sendo 57,1% do sexo masculino, 81,0% não brancos e com mediana (intervalo interquartil) de idade ao diagnóstico de seis (2–42) meses. Havia 21, 23 e 19 indivíduos nos subgrupos 1, 2 e 3, respectivamente. Ao diagnóstico, 26 (41,3%) indivíduos apresentavam baixa estatura (BE) ou muito baixa estatura (BE), correspondendo a 57,1, 43,5 e 21,1% das crianças nos subgrupos 1, 2 e 3, respectivamente. Após um ano, essas proporções diminuíram para 28,5 e 30,4% nos subgrupos 1 e 2, enquanto permaneceram inalteradas no subgrupo 3. A insuficiência pancreática (IP) estava presente em 85,7, 69,6 e 68,4% dos participantes nos subgrupos 1, 2 e 3, respectivamente.

**Conclusões:**

A baixa estatura foi frequente ao diagnóstico, mesmo entre crianças identificadas pela triagem neonatal. Após um ano de acompanhamento, a estatura melhorou nos subgrupos 1 e 2, mas permaneceu inalterada no subgrupo 3. O diagnóstico precoce e as intervenções nutricionais oportunas são essenciais para prevenir e corrigir os déficits de crescimento em indivíduos com FC.

## INTRODUCTION

 Cystic fibrosis (CF) is an autosomal recessive genetic disorder caused by pathogenic variants in the *CFTR* (Cystic Fibrosis Transmembrane Conductance Regulator) gene, which encodes the CFTR, a membrane protein responsible for chloride and bicarbonate transport across epithelial cells. Dysfunction or absence of CFTR leads to thick secretions that primarily affect the respiratory and gastrointestinal systems, resulting in chronic lung disease, pancreatic insufficiency (PI), and nutritional impairment.^1-3^

 Early diagnosis of CF is essential to reduce morbidity and improve long-term outcomes. Diagnosis may be established through newborn screening (NBS) or based on clinical manifestations, with confirmation by sweat testing and/or molecular analysis of *CFTR*.^4^ Evidence from international studies consistently demonstrates that NBS enables earlier diagnosis and timely initiation of treatment, including pancreatic enzyme replacement therapy and nutritional support, which are associated with improved growth, better nutritional status, and reduced hospitalizations.^5-7^

 Historically, CF diagnosis was often delayed until the onset of clinical signs such as failure to thrive, malnutrition, or recurrent respiratory infections. The implementation of NBS programs has substantially changed this scenario in many countries. In Brazil, NBS for CF was introduced progressively, beginning in 2001 in Paraná and later expanding nationwide.^8^ In Bahia, CF screening was incorporated into the national newborn screening program in 2013.^9^ Despite these advances, regional disparities in screening coverage, diagnostic confirmation, and access to specialized care persist, which may influence clinical and nutritional outcomes. 

 Nutritional status is a key determinant of prognosis in CF and is closely associated with pulmonary function, survival, and quality of life.^10-12^ Although early nutritional interventions have improved outcomes, children with CF may still present growth deficits compared with healthy peers, particularly when diagnosis and treatment are delayed.^13,14^ These challenges may be intensified in low- and middle-income settings, where socioeconomic factors and healthcare inequities affect access to timely diagnosis and specialized follow-up. 

 While international studies have demonstrated nutritional advantages among children diagnosed through NBS, Brazilian data comparing nutritional outcomes according to diagnostic method remain limited, especially considering regional differences in healthcare access. This gap restricts a comprehensive understanding of the impact of NBS on nutritional outcomes in Brazilian children with CF. 

 Therefore, this study aimed to compare the nutritional status of children with CF diagnosed through NBS or based on clinical findings at the time of diagnosis and after 1 year of follow-up. 

## METHOD

 This is an observational study. A retrospective stage began with the first consultation (admission) between January 2005 and January 2012. Prospective follow-up occurred from February 2012 to December 2023. Individuals treated at the interdisciplinary cystic fibrosis outpatient clinic (ICFOC) at a University Referral Center in Salvador were monitored during this time. All consecutive CF individuals were invited to participate in the study. In the state of Bahia, there are only two specialized centers for CF care, both located in Salvador, the state capital. All individuals with CF are referred to and followed up at one of these centers. 

 The inclusion criteria were a confirmed CF diagnosis (elevated sweat chloride on two occasions and/or identification of two pathogenic variants in the *CFTR* gene) and regular follow-up at the ICFOC for at least 1 year after diagnosis. Individuals who presented comorbidities that could lead to nutritional impairment, such as hepatic diseases, type 1 diabetes mellitus, and congestive heart failure, were excluded. 

 The study variables included demographic, genetic, clinical, and anthropometric data. Demographic variables comprised sex, race/ethnicity, current age, and age at diagnosis. Genetic variables included *CFTR* genotype, classified according to residual or non-residual protein function. Clinical variables included the diagnostic pathway (newborn screening or clinical signs and symptoms), pancreatic insufficiency status, fecal elastase-1 results, and the use of Enzyme Replacement Therapy (ERT) when the test was unavailable. Anthropometric variables included weight, height, and body mass index (BMI) measured at diagnosis and after 1 year of follow-up. Z-scores for height-for-age (H/A) and BMI-for-age (BMI/A) were calculated according to World Health Organization (WHO) reference standards.^15^

 The ICFOC operates weekly, and at each consultation, nutritionists perform a nutritional assessment. This assessment includes a nutritional history and anthropometric measurements, followed by continuous, individualized nutritional intervention. 

 At the beginning of the nutritional assessment, children under 2 years of age were weighed without clothes on a Filizola® digital pediatric scale (São Paulo, Brazil), and those over 2 years of age were weighed wearing only underwear on a Welmy® platform scale (São Paulo, Brazil). Height measurements for children under 2 years of age were performed by placing the child in the center of the infantometer. Participants aged over 2 years were measured standing, using a stadiometer with a scale in centimeters and an accuracy of 1 mm. 

 Anthropometric data were entered and evaluated using the WHO Anthro application version 3.2.2, 2011, for children aged 0–4 years and 11 months, and the WHO Anthro Plus version 1.0.4, 2007, for children aged 5–19 years. Z-scores for H/A, BMI/A, and Weight/Age (W/A) were calculated for children under 10 years. For children aged 10 years or older, only H/A and BMI/A were calculated.^15^

 Regarding race/ethnicity, participants were classified as white or non-white by the coordinating researcher at the study’s reference center, based on the child’s phenotypic characteristics. The diagnosis of PI was primarily defined as fecal elastase-1 <200 μg/g. However, for individuals for whom the test was unavailable at the referral center, PI classification was based on clinical evidence of malabsorption and on ERT use. 

 Based on participants’ genotypes, CFTR protein functionality was classified as: no residual function when individuals had two variants in the *CFTR* gene of classes I–III, and residual function when they had at least one variant in classes IV–VI. Genotypes with no residual function indicate an inactive or absent CFTR protein, which is usually associated with more severe phenotypes. In contrast, genotypes with residual function show some CFTR activity, although usually reduced, leading to less severe symptoms.^16^

 SPSS version 18 (IBM SPSS Statistics) was used for data analysis and tabulation. Quantitative variables were described using the mean and standard deviation or the median and interquartile range (IQR), depending on the variable’s distribution. Categorical variables were described using simple, absolute, and relative frequencies. 

 Based on diagnostic criteria and the participants’ ages, individuals were classified into three subgroups: Subgroup 1: Diagnosis based on NBS; Subgroup 2: Diagnosis based on signs and symptoms before 24 months of age (early signs and symptoms); Subgroup 3: Diagnosis based on signs and symptoms after 24 months of age (late signs and symptoms). BMI/A and H/A Z-scores among the three subgroups were compared using the Kruskal–Wallis test or ANOVA (one-way analysis of variance), depending on the distribution of the variable. Mann-Whitney and Tukey tests were used for post-hoc analysis, respectively. To compare these variables at diagnosis and 1 year later, the Wilcoxon test or the t-test for dependent samples was used. A p<0.05 was considered statistically significant. 

 Based on the BMI/A results, a variable describing the time course of this indicator in each subgroup during the follow-up period was created. A positive effect was considered when the individual showed improvement or maintained a normal BMI/A. Conversely, a negative effect was considered when the individual’s nutritional status worsened. 

 This study is part of a broader project approved by the Institutional Ethics and Research Committee under opinion number 7.106.037 and under the Certificate of Presentation for Ethical Consideration (CAAE) number 58704316.0.0000.0049. 

## RESULTS

**Figure 1:**
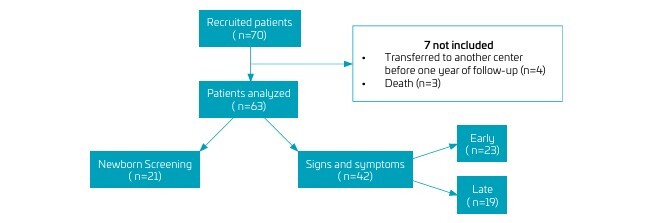
Flowchart of patient selection.

 Seventy individuals were recruited, of whom seven were not included, four were transferred to another center before completing 1 year of follow-up, and three died before completing 1 year in the study. This left 63 individuals for analysis (Figure 1). The median (IQR) age of participants was 6 (2–42) months. Most (57.1%) were male, and 51 (81.0%) were nonwhite. Demographic characteristics and the occurrence of PI at diagnosis in the subgroups studied are presented in Table 1. PI occurred in 47 (75%) of the individuals, being more common in subgroup 1 (86% of the participants). 

**Table 1:** Demographic characteristics and occurrence of pancreatic insufficiency at diagnosis in subgroups of individuals with cystic fibrosis from the university reference center, Brazil, 2005–2023.

Variable	Diagnostic classification		
	Subgroup 1 (newborn screening) 21 (33.3%)	Subgroup 2 (early signs and symptoms) 23 (36.5%)	Subgroup 3 (late signs and symptoms) 19 (30.2%)
Current age (in months)*	17 (15–17)	21 (18–23)	82 (62–182)
Mean age at diagnosis (months)¹	2 (1–3)	6 (5–7)	64 (43–163)
Male sex	11 (52.4)	12 (52.2)	13 (68.4)
Non-white race†	16 (76.2)	20 (87.0)	15 (78.9)
Rural residence	15 (71.4)	11 (47.8)	13 (68.4)
Pancreatic insufficiency	18 (85.7)	16 (69.6)	13 (68.4)

Notes: Current age=Age after 12 months from diagnosis.

*Median (Interquartile range); †Non-white race=mixed people+black.

Table 2 describes the nutritional course of the participants in each subgroup. At diagnosis, short stature (SST) or very SST was observed in 41.3% of the total sample, with marked differences among diagnostic subgroups. The highest frequency occurred in subgroup 1 (57.1%), followed by subgroup 2 (43.5%), whereas subgroup 3 showed the lowest prevalence (21.1%). Despite the earlier diagnosis, children identified through newborn screening already presented substantial linear growth impairment at baseline. Comparative analysis of height-for-age (H/A) Z-scores demonstrated significant differences between subgroups at diagnosis (p=0.008), with subgroup 2 presenting the lowest mean values. After 1 year of follow-up, significant improvements in H/A were observed in subgroups 1 and 2, and intergroup differences were no longer statistically significant (p<0.001 for within-group improvement; p=0.091 between groups). 

**Table 2:** Nutritional status evolution of patients according to the type of cystic fibrosis diagnosis at a university reference center Brazil, 2005–2023.

Variable	Cystic fibrosis diagnosis groups						p-value	
	Subgroup 1 (newborn screening) 21 (33.3%)		Subgroup 2 (early signs and symptoms) 23 (36.5%)		Subgroup 3 (late signs and symptoms) 19 (30.2%)			
	At diagnosis	1 year after	At diagnosis	1 year after	At diagnosis	1 year after	At diagnosis	1 year after
H/A Z-score*	**−2.39 (1.84)**	**-1.14 (1.35)**	**-2.64 (2.30)**	**-0.93 (1.66)**	**-1.15 (1.04)**	**-0.93 (0.98)**	**0.051†**	**0.733‡**
p-value between groups	**0.008§**		**<0.001//**		**0.091¶**			
H/A Z-score Classification (%)								
Very short height-for-age	8 (38.1)	2 (9.5)	8 (34.8)	3 (13.0)	1 (5.3)	-		
Short height-for-age	4 (19.0)	4 (19.0)	2 (8.7)	4 (17.4)	3 (15.8)	4 (21.1)		
Appropriate height-for-age	9 (42.9)	15 (71.4)	13 (56.5)	16 (69.6)	15 (78.9)	15 (78.9)		
BMI/A Z-score*	**-0.98 (1.22)**	**0.01 (1.13)**	**-1.48 (1.55)**	**0.23 (1.27)**	**-0.91 (1.06)**	**-0.44 (0.84)**	**0.305†**	**0.212‡**
p-value between groups	**0.013§**		**<0.001//**		**0.035¶**			
Classification BMI/A Z-score (%)								
Underweight	2 (9.5)	-	5 (21.7)	-	3 (15.8)	-		
Normal nutritional status	18 (85.7)	18 (85.7)	17 (73.9)	17 (73.9)	16 (84.2)	17 (89.5)		
Overweight	1 (4.8)	3 (14.3)	1 (4.3)	6 (26.1)	-	2 (10.5)		

*Mean and standard deviation of Z score; †ANOVA test comparing subgroups 1, 2, and 3 at diagnosis; ‡ANOVA test comparing subgroups 1, 2, and 3 one year after treatment; §Kruskal-Wallis test comparing within-group subgroup 1 at diagnosis and one year after treatment; //Kruskal-Wallis test comparing within-group subgroup 2 at diagnosis and one year after treatment.; ¶Kruskal-Wallis test comparing within-group subgroup 3 at diagnosis and one year after treatment.;

BMI/A: Body Mass Index-for-Age; H/A: Height-for-Age.

Bold values indicate statistically significant results (p<0.05).

**Figure 2:**
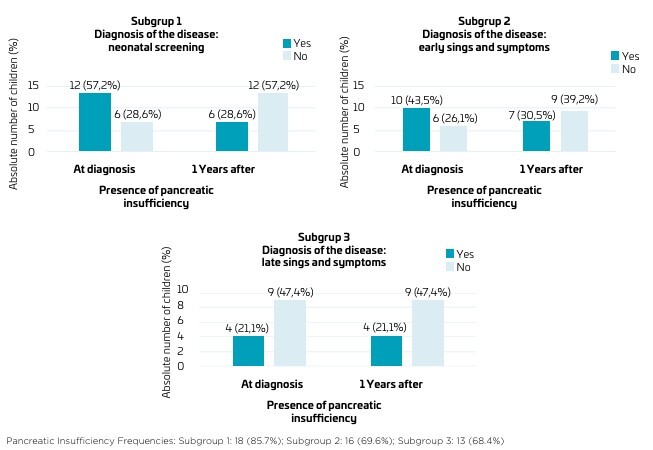
Evolution of short stature in individuals with pancreatic insufficiency across subgroups.

 Among individuals with pancreatic insufficiency, 55.3% presented SST at diagnosis. After 1 year of follow-up, this proportion decreased to 36.2%, indicating partial recovery of linear growth in early diagnosed groups. Regression of SST occurred in 50.0% of children in subgroup 1 and in 30.0% of those in subgroup 2. In contrast, although SST was less frequent in subgroup 3 at diagnosis, no regression of this condition was observed during the follow-up period (Figure 2). 

**Figure 3:**
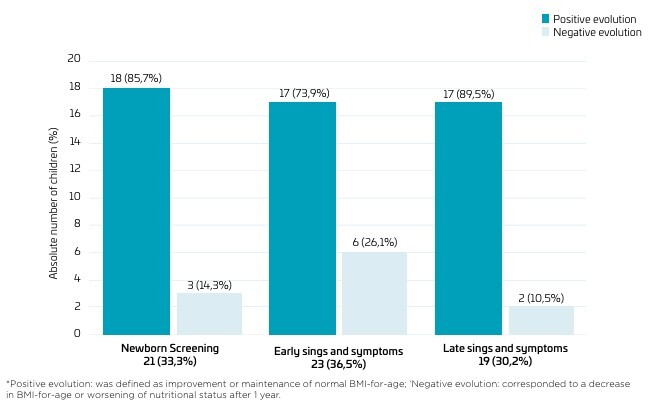
Body mass index-for-age evolution after 1 year of follow-up according to conditions at diagnosis.

 Regarding body mass index-for-age (BMI/A), no significant differences in nutritional status were observed between subgroups at diagnosis. However, subgroup 2 presented lower mean BMI/A Z-scores, and between-group comparisons revealed statistically significant differences at baseline (p=0.013). After 1 year of follow-up, BMI/A improved across all subgroups, with most individuals achieving or maintaining adequate nutritional status. Nevertheless, intergroup differences persisted after follow-up (p=0.035), reflecting heterogeneous nutritional responses to treatment (Table 2). Analysis of BMI/A trajectory showed a favorable evolution in 85.7% of individuals in subgroup 1, 73.9% in subgroup 2, and 89.5% in subgroup 3. Notably, subgroup 2 presented the highest proportion of negative BMI/A evolution (26.1%), suggesting greater difficulty in sustaining nutritional recovery over time (Figure 3). 

 Genotypic analysis demonstrated a higher frequency of CFTR variants associated with no residual protein function in subgroups 1 and 2 compared to subgroup 3. This distribution is consistent with the higher prevalence of pancreatic insufficiency and the greater severity of growth impairment observed at diagnosis in the earlier-diagnosed groups (Table 3). 

**Table 3.** Genotypes of participants according to Cystic Fibrosis Transmembrane Conductance Regulator protein function in the three subgroups at a university reference center, Brazil, 2005–2023.

Genotype		Cystic fibrosis diagnosis		
		Subgroup 1 (newborn screening) 21 (33.3%)	Subgroup 2 (early signs and symptoms) 23 (36.5%)	Subgroup 3 (late signs and symptoms) 19 (30.2%)
Residual function in the CFTR protein				
	No residual function*	13 (61.9)	14 (60.9)	9 (47.4)
	With residual function†	3 (14.3)	3 (13.0)	2 (10.5)
	Unclassified‡	5 (23.8)	6 (26.1)	6 (31.6)
	Only one pathogenic variant identified	-	-	2 (10.5)

CFTR: Cystic Fibrosis Transmembrane Conductance Regulator.

*Presence of two cystic fibrosis-causing variants of classes I–III; †Presence of at least one variant of classes IV–VI; ‡Individuals with one or more unclassified variants.

## DISCUSSION

 This study demonstrated a high frequency of SST at diagnosis, particularly in subgroups 1 and 2. At baseline, no significant differences in BMI impairment were observed between the subgroups, although height-for-age was lower, especially in subgroup 2. After 1 year of follow-up, a reduction in SST was observed in subgroups 1 and 2, whereas in subgroup 3 the frequency remained unchanged, suggesting limited recovery of linear growth in individuals diagnosed later. These findings reinforce the importance of early diagnosis and timely nutritional and therapeutic interventions for growth recovery in CF. 

 Evidence indicates that children with CF who do not undergo NBS are at increased risk of growth impairment due to delayed diagnosis and late initiation of treatment.^13,14,17^ In the present study, despite earlier diagnosis, children in subgroup 1 already showed marked nutritional impairment, likely related to the high prevalence of PI, a condition that compromises nutrient digestion and absorption and contributes to growth deficits.^18,19^ Additionally, although this group was diagnosed through NBS, the median age at diagnosis was 2 months, indicating delays in confirmatory diagnosis and referral to specialized care.^7,20^

 The positive impact of NBS is also associated with the potential for early intervention and improved clinical outcomes, including better growth and quality of life.^5,6^ As evidenced by Wagener et al.^6^ and Martins et al.,^21^ children diagnosed in the first months of life are more likely to achieve weight and height closer to the ideal, reducing the impacts of CF on child growth. In fact, in this study, although subgroup 1 had the highest percentage of individuals with SST, after 1 year of follow-up, 50% had adequate height for their age, reinforcing the importance of specialized care.^21^

 Part of the severity observed in this subgroup may be associated with genotype, as a significant proportion of patients had *CFTR* variants with no residual function, which are linked to more severe clinical conditions.^16^ In addition to genetic characteristics, delays in starting specialized treatment and difficulties faced by families in dealing with the routine care of a chronic disease also negatively influence clinical outcomes, especially in regions with weaknesses in the NBS program.^22,23^

 Previous studies show that implementing appropriate nutritional strategies, including specialized dietary support and ERT, can significantly improve patient clinical outcomes, reducing the risk of malnutrition and its associated complications.^11,21,24^ In the present study, children classified in subgroup 2 had greater H/A impairment at diagnosis, and during follow-up, the BMI/A variable had a higher rate of negative evolution; 26% of individuals showed worsening, suggesting greater challenges in treatment and nutritional recovery and/or that the intervention applied was less effective compared to the others. It should be noted that several other variables can influence nutritional outcomes, such as adherence to treatment and socioeconomic conditions, which were not evaluated in the present study. 

 Despite fewer individuals and later diagnoses, subgroup 3 showed less nutritional impairment at the time of diagnosis. However, when SST was present, it did not reverse during follow-up. These findings suggest that individuals in this subgroup had milder clinical forms of CF, consistent with the lower frequency of both *CFTR* gene variants with no residual function^16^ and the PI, compared to the other subgroups. Although they showed greater positive changes in BMI/A, these did not translate into height gains, indicating that the timing of diagnosis and early initiation of treatment are determining factors in the recovery of linear growth.^14,21,25^

 While weight gain can occur more rapidly, linear growth requires longer follow-up and may be persistently or even irreversibly impaired due to the chronic nature of the disease.^1,3^ It is important to note that NBS for CF was only implemented in the state of Bahia in 2013, and false-negative results are possible.^8,9^ This reinforces the critical role of primary care in active surveillance, qualified clinical assessment, and coordination with other levels of care to ensure timely diagnosis. In this context, clinical suspicion remains essential, especially in cases not detected by NBS, and ongoing training of primary healthcare professionals is indispensable to promote early interventions, prevent complications, and improve the quality of life of children with CF and their families. 

 Although CF is more prevalent in populations of Caucasian origin, this study identified a high frequency of the disease among non-white individuals across all three subgroups. This reflects the diverse ethnic composition of the Brazilian population, shaped by the miscegenation of Indigenous, African, and European peoples.^9,26^ This diversity influences the genetic profile of Brazilian patients, characterized by a greater heterogeneity of *CFTR* variants, including rare and novel mutations.^26,27^ These variations may impact the clinical phenotype and complicate early diagnosis in the absence of NBS, as possibly observed in subgroup 3. 

 PI is a central manifestation of CF and, if untreated, is a major cause of nutritional impairment, particularly in relation to height. In an experimental model, Schubert et al.^19^ demonstrated that PI leads to significant morphological changes in the intestine, impairing nutrient digestibility and directly affecting growth parameters. These findings underscore the importance of timely diagnosis and treatment of PI to prevent SST and improve clinical and nutritional outcomes in children with CF. In the present study, the frequency of PI was 85.7, 69.6, and 68.4% in subgroups 1, 2, and 3, respectively. An improvement in height and a reduction in SST were observed in subgroups 1 and 2, which was not the case in subgroup 3, where the initial distribution remained unchanged. 

 This study has some limitations. Because it was a single-center study, the findings cannot be generalized to other populations or regional contexts, given the specific characteristics of the study group and its small population. The small sample size may not have captured the full clinical variability of individuals with CF, and the short follow-up period (1 year) restricts the evaluation of long-term nutritional outcomes. Another limitation is the absence of systematic information on treatment adherence and socioeconomic conditions. However, previous analyses of the same population indicated that most families are low-income and live in rural areas. These factors are known to influence nutritional status and clinical outcomes in CF, may hinder access to specialized services typically concentrated in capital cities, and could have contributed to the variability observed in this cohort, thereby limiting the interpretation of their potential impact on the results.^23^ An additional limitation was the definition of PI, which in part of the sample relied on clinical findings and use of ERT rather than fecal elastase-1, possibly leading to misclassification. However, nearly 70% of these patients carried genotypes with two variants lacking residual CFTR function, strongly associated with PI. 

 Despite its limitations, this study provides relevant insights into the nutritional outcomes of individuals with CF diagnosed at different ages, reinforcing the importance of personalized care. Early-diagnosed patients, especially those identified through NBS, showed improvement or stabilization of nutritional status after 1 year, despite greater initial impairment. These findings emphasize the value of timely diagnosis, specialized follow-up, and continuous reassessment of NBS programs, as well as the need to educate pediatricians and primary care professionals to reduce diagnostic delays. In Brazil, the restricted access to CFTR modulators further highlights the urgency of optimizing all stages of diagnosis and care to improve long-term prognosis. 

 Beyond ensuring earlier diagnosis, the data also highlight the need to further strengthen the CF NBS program by incorporating early, personalized intervention strategies and continuous monitoring that consider genetic, nutritional, and socioeconomic factors. Specialized CF care centers play a crucial role in modifying the natural history of the disease, minimizing complications, and improving long-term prognosis. 

 This study demonstrated that SST was frequent at diagnosis, including among children identified through NBS. After 1 year of follow-up, significant improvements were observed in the early-diagnosed subgroups, whereas late-diagnosed patients continued to show growth deficits. These findings underscore the importance of early diagnosis and timely interventions, including nutritional support, to prevent and correct growth impairments, reinforcing the need to strengthen NBS programs and ensure comprehensive multidisciplinary care to improve long-term outcomes and quality of life in children with CF. 

## Data Availability

The database that originated the article is available with a corresponding author.
